# Genome-Wide Association Mapping of QTL Underlying Groat Protein Content of a Diverse Panel of Oat Accessions

**DOI:** 10.3390/ijms24065581

**Published:** 2023-03-15

**Authors:** Honghai Yan, Haixu Zhang, Pingping Zhou, Changzhong Ren, Yuanying Peng

**Affiliations:** 1 State Key Laboratory of Crop Gene Exploration and Utilization in Southwest China, Sichuan Agricultural University, Chengdu 611130, China; honghai_yan@outlook.com; 2 Triticeae Research Institute, Sichuan Agricultural University, Chengdu 611130, China; 3 National Oat Improvement Center, Baicheng Academy of Agricultural Sciences, Baicheng 137000, China

**Keywords:** oat, groat protein content, GWAS, favorable alleles

## Abstract

Groat protein content (GPC) is a key quality trait attribute in oat. Understanding the variation of GPC in oat germplasms and identifying genomic regions associated with GPC are essential for improving this trait. In this study, the GPC of 174 diverse oat accessions was evaluated in three field trials. The results showed a wide variation in GPC, ranging from 6.97% to 22.24% in this panel. Hulless oats displayed a significantly higher GPC compared to hulled oats across all environments. A GWAS analysis was performed based on 38,313 high-quality SNPs, which detected 27 non-redundant QTLs with 41 SNPs significantly associated with GPC. Two QTLs on chromosome 6C (QTL16) and 4D (QTL11) were consistently detected in multiple environments, with QTL16 being the most significant and explaining the highest proportion of the phenotypical variation in all tested environments except in CZ20. Haplotype analysis showed that the favorable haplotypes for GPC are more prevalent in hulless oats. These findings provide a foundation for future efforts to incorporate favorable alleles into new cultivars through introgression, fine mapping, and cloning of promising QTLs.

## 1. Introduction

Oat (*Avena sativa* L.), belonging to the genus *Avena* L. of the grass family is the sixth most important cereal crop globally, after rice, wheat, maize, barley, and sorghum [1]. In recent years, oats have become increasingly popular due to their many health benefits. Oats are a rich natural source of antioxidants, proteins, and dietary fiber [2]. Consumption of oats has been shown to lower blood cholesterol levels, thereby reducing the risk of cardiovascular diseases [3,4]. Over the past decades, oat yield has increased steadily with the development and introduction of new high-yield cultivars. In addition to yield, modern cultivars are also expected to have high quality, such as high β-glucan and protein content. However, most efforts to improve oat cultivars for food focused primarily on increasing the β-glucan content [5], while there has been little emphasis on increasing the groat protein content (GPC).

GPC is an important trait for both nutritional value and end-use quality of oats [6]. Oat groat possesses the highest protein levels among cereal crops, with levels ranging from 15 to 21% in the de-hulled kernel [7,8]. Unlike other temperate cereals such as wheat, barley, and rye, which have a high percentage of prolamins, oat proteins are mainly composed of globulins (50–80%) [9] and contain no gluten and only a low level of gluten-related prolamins, making them a safe food for people with gluten intolerance [2,10]. In addition, oat protein has a well-balanced amino content, which varies little with changes in protein content [11]. Agronomic and genetic biofortification are two methods that can increase the GPC in oats. Agronomic biofortification involves the use of nitrogen fertilizer [12,13], but it increases cost and environmental pollution. In contrast, genetic GPC biofortification is likely to be more cost-effective in the long term. This requires the existence of genetic variation in oat germplasm. Over 80,000 oat accessions are maintained in world Genebanks [14]; however, limited information is available on the variation in GPC among oat genotypes. In a previous study, Brown et al. [7] determined the GPC of 129 spring and 40 winter oat genotypes grown in Illinois using the standard Kjeldahl method and found a variation in GPC ranging from 15.2% to 20.8%. More recently, the GPC of 50 oat accessions stored at the N.I. Vavilov Institute for Plant Genetic Resources (VIR) was determined. These lines showed a variation in GPC of 10.9% to 19.3% [15]. In recent years, there has been a growing interest globally in hulless oats due to the absence of thick and fibrous hulls. Nevertheless, limited research has been conducted to investigate the variation in GPC of hulless oats.

The identification of genetic loci associated with GPC is crucial for improving this trait in oats. The first quantitative trait locus (QTL) analysis for oat GPC was conducted by Zhu et al. [16] using the ‘Ogle’/‘MAM17-5’ mapping population. This analysis identified 17 QTLs located on 13 linkage groups, explaining 29–42% of the total phenotypic variation in two years. Subsequent QTL analyses using recombinant inbred line (RIL) mapping populations identified three QTLs for GPC in the ‘Terra’/’Marion’ mapping population and linkage map [17] and five QTLs in the ‘Aslak’/‘Matilda’ mapping population and linkage map [18]. Additionally, Hizbai et al. [19] and Herrmann et al. [20] reported one and two QTLs, respectively, associated with GPC in oat. Of these QTLs identified, a single locus could explain up to 42% of the phenotypic variation [16].

Despite some genomic regions associated with GPC in oats having been identified previously, most of the genetic maps used in these studies have been generated using diverse DNA markers. The paucity of common markers among these maps has made the comparisons of the positions of QTLs challenging [19]. Moreover, most of these studies used bi-parental mapping populations to identify the QTLs associated with oat protein content. As a result, the association between markers and QTL may be significant in only specific genetic backgrounds due to low mapping resolution [3].

Association mapping is a powerful alternative to traditional QTL mapping approaches. It identifies QTLs by utilizing the majority of recombination events of a set of diverse germplasms that have occurred in the evolutionary history, resulting in higher mapping resolution compared to linkage mapping [21]. Several GWAS analyses have been conducted on various agronomic traits in oats, including β-glucan content [3,22,23], heading date [24], crown rust resistance [25], seed vigor [26], and hulless grain [27,28]. These studies not only confirmed QTLs that were previously detected by linkage mapping, but also identified many new ones, thus providing valuable information for oat breeding and genomic research.

Recent advancements in oat genomics have led to the generation of some high-quality and fully annotated oat genomes [2,28]. These genomic resources hold great potential for oat genomic studies [2,5,28]. In a previous study, we generated a chromosome-level reference genome for the oat variety “Sanfensan” [28]. Subsequently, a GWAS was conducted by using 49,702 high-quality SNPs identified through mapping the genotyping-by-sequencing (GBS) data from 659 diverse oat varieties to the reference genome. This allowed us to identify a candidate region associated with the hulless grain trait. Although GPC is a major quality trait in oat, to our knowledge, no previous GWAS has been performed to identify markers associated with this trait. In this sense, the main goals of this study were to evaluate the GPC in a panel of 174 diverse oat accessions through multiple-environment assessments and to identify genomic regions associated with this trait using GWAS, which can ultimately aid in the improvement of oat quality through molecular-assisted selection.

## 2. Results

### 2.1. Variation in Groat Protein Content among Diverse Oat Accessions

**Figure 1:**
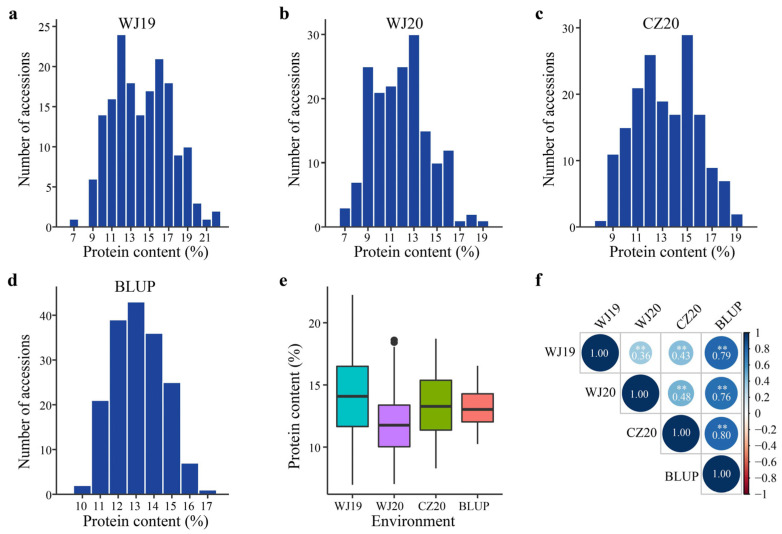
Distribution of the groat protein contents of the 174 diverse oat accessions. Distribution of the groat protein contents at Wenjiang during the 2018–2019 (WJ19) (**a**) and 2019–2020 (WJ20) (**b**) cropping seasons, at Chongzhou in 2019–2020 (CZ20) (**c**), and in multi-environment model (**d**). BLUP in (**d**) represents the best linear unbiased prediction. (**e**) Boxplot of the groat protein content in the individual environments (n = 174 independent oat accessions). The central line for each box plot indicates the median. The white diamond represents the mean value. The top and bottom edges of the box indicate the first and third quartiles and the whiskers extend 1.5 times the interquartile range beyond the edges of the box. The black dots represent the outliers. (**f**) Pearson’s correlation coefficients (r) of pairwise environments. Numbers in the circles indicate the r values. The asterisks represent significant differences (two-tailed Student’s *t*-test, **, *p* < 0.01).

The GPC of 174 diverse oat accessions was evaluated on a dry groat weight basis in three field trials. A wide variation of GPCs, ranging from 6.97% to 22.24% across three trials, was observed, which is in line with the segregation of displaying quantitative traits (Table S1, Figure 1a–d). The highest mean GPC (14.25%) was recorded in Wenjiang during the 2018–2019 cropping season (WJ19), whereas the lowest mean GPC was in Wenjiang during the 2019–2020 cropping season (WJ20) (Table 1, Figure 1e). Pearson’s correlation coefficient showed a significant correlation across environments (Figure 1f). Analysis of variance revealed significant effects of genotype and environment on GPC (Table 2).

**Figure 2:**
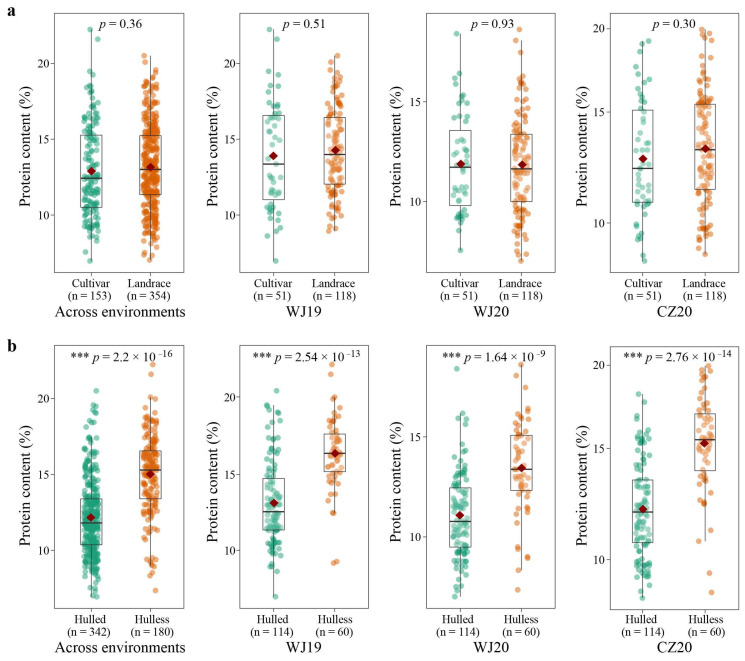
Comparison of protein contents between cultivars and landraces (**a**); hulled and hulless oats (**b**) across three environments and in individual environments. The central line of each box plot represents the median. The top and bottom edges of the box indicate the first and third quartiles, and the whiskers extend 1.5 times the interquartile range beyond the edges of the box. The red diamond indicates the mean value. A two-tailed Student’s *t*-test was used to generate the *p* values (*** *p* < 0.001).

Across three environments, the 51 modern cultivars exhibited a mean GPC of 12.89%, ranging from 6.97 to 22.24%, which was lower than that of the landraces (13.17%) (Figure 2a, Table S2). However, the difference was not statistically significant (*p* = 0.36). This trend held true in each environment except in WJ20, where the mean GPC of cultivars was slightly higher than that of landraces (11.89% vs. 11.85%) (Figure 2a). The 60 hulless oats had a significantly higher GPC than the 114 hulled oats across all three environments (15.02% vs. 12.16%, *p* < 0.001) and in each individual environment (Figure 2b, Table S2). Except for a significantly higher GPC of hulled landraces than that of hulled cultivars in CZ20, no significant differences (*p* > 0.05) were found between modern cultivars and landraces of either hulled or hulless oats in the testing environments (Figure 2c,d).

The screening of the 174 diverse oat accessions for consistent high (top 20) and low (bottom 20) GPC in at least two environments identified 11 accessions with high GPC and eight accessions with low GPC (Table 3). Of the 11 high GPC accessions, all were hulless oats, with six being landraces and four being cultivars, whereas of the eight low GPC oats, six were hulled oats.

### 2.2. Marker-Trait Associations for Protein Content

**Figure 3:**
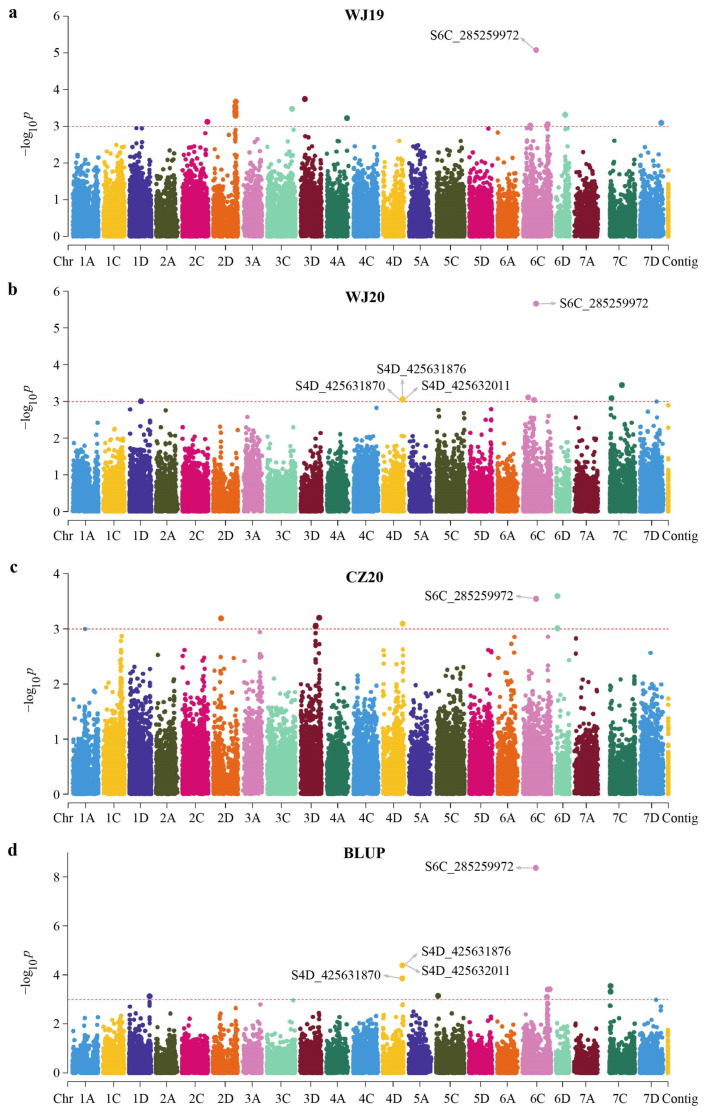
Manhattan plots of genome-wide association scan for oat groat protein content in the 174 diverse oat accessions in different environments using the mixed linear model (MLM) in TASSEL. Each dot represents an SNP. The horizontal dashed line represents the significant threshold −log_10_(*p*) equaling 3.0. The SNPs above the red dotted line are all significantly associated with the groat protein content. (**a**) WJ19, (**b**) WJ20, (**c**) CZ20, (**d**) multi-environment model. Names of significant markers detected in multiple environments are highlighted.

**Figure 4:**
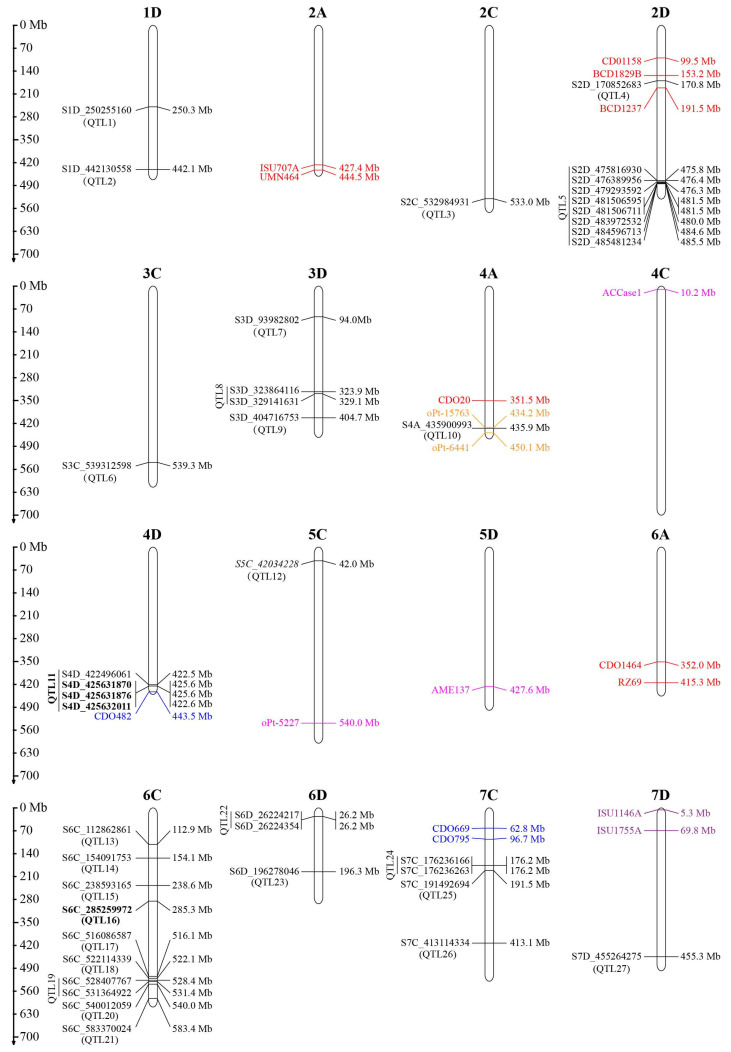
Chromosomal locations of QTLs for oat groat protein content identified by previous studies and in this study. The blue, red, pink, and purple names represent QTL identified by Zhu et al. [16], De Koeyer et al. [17], Tanhuanpää et al. [19], and Hizbai et al. [18], respectively. The QTLs identified in this study are represented in black, and the two QTLs detected in multiple environments are highlighted in bold.

A mixed linear model accounting for the population structure and familial relationship was used for association analysis (Figure 3). A total of 41 SNP markers were identified to be significantly associated (*p* < 1 × 10^−3^) with GPC based on phenotypic data in individual environments and BLUP values across three environments (Table S3). These markers could be grouped into 27 non-redundant QTLs distributed on 12 chromosomes based on their chromosome positions (Table 4, Figure 4). In WJ19, 11 QTLs comprised of 19 SNPs were detected and located on 2C, 2D, 3C, 3D, 4A, 6C, 6D, and 7D. These SNPS explained 10.18–17.81% of the phenotypic variation. In WJ20, nine associated markers belonging to seven QTLs were identified. These markers were located on 1D, 4D, 6C, and 7C, and explained 10.14 to 20.00% of the total phenotypic variation. Similarly, eight SNPs distributed on 2D, 3D, 4D, 6C, and 6D were identified to be significantly associated with GPC in CZ20. These markers explained 8.30–11.92% of the phenotypic variation. Eleven significant associated SNPs distributed on 1D, 4D, 5C, 6C, and 7C were detected in the multi-environment model, explaining 10.35–30.58% of the phenotypic variation.

Among the 27 QTLs, two QTLs (QTL11 and QTL16) were detected in at least two environments including BLUP (Table 4). QTL11 was comprised of four markers, while QTL16 was represented by one marker. SNP marker S6C_285259972 located at 285.3 Mb was linked with QTL16 on 6C. This marker was consistently detected in all environments (including BLUP) and explained the highest phenotypic variation (17.81–30.58%) in all environments except in CZ20, where it explained 11.75% (second highest) of the phenotypic variation. Four markers, S4D_425631870, S4D_425631876, S4D_425632011, and S4D_422496061 were associated with QTL11 on 4D. Of these, S4D_425631870, S4D_425631876, and S4D_425632011 were detected in WJ20 and in the multi-environment model, explaining 10.46–15.38% of the phenotypic variation. These SNPs could be considered as stable loci for the regulation of GPC in oats.

### 2.3. Comparison to Previous QTL Studies

The chromosomal locations of previously reported QTLs [16,17,18,19,20] were determined by aligning the flanking markers to the “Sanfensan” reference genome. Of the 24 QTLs reported previously, 12 were located unambiguously on the chromosomes (Figure 4). A comparison of the loci identified in the present study with these known QTLs showed that two QTLs located on chromosomes 2D (QTL4) and 4A (QTL10) overlapped with the previously known QTLs. In addition, QTL11 on chromosome 4D (422.5–425.6 Mb) was located near a QTL on the TM_5 linkage group reported by De Koeyer et al. [17] (Figure 4).

### 2.4. Favorable Haplotype Analyses

**Figure 5:**
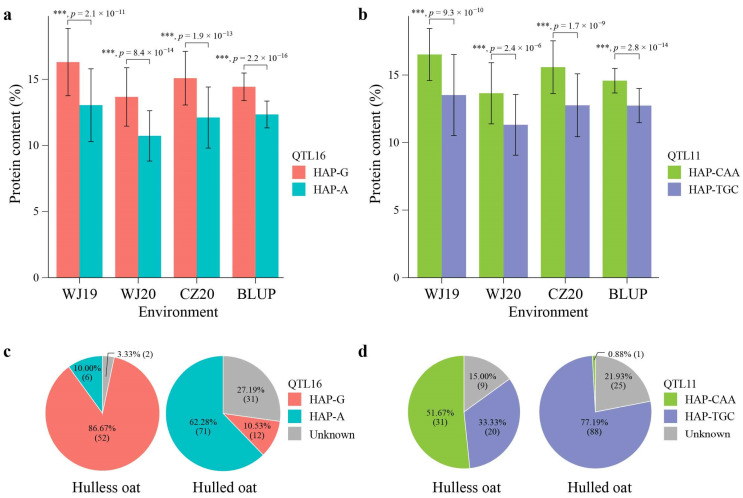
Genetic effects of bi-allele variation at QTL16 (**a**) and QTL11 (**b**) in relation to groat protein content in different environments, and allele frequencies of QTL16 (**c**) and QTL11 (**d**) in hulless and hulled oats. n = 77, with 64 independent oat accessions for HAP-G and HAP-A in a, and n = 108, 32 independent oat accessions for HAP-CAA and HAP-TGC in b, respectively. A two-tailed Student’s *t*-test was used to generate the *p* values (***, *p* < 0.001).

The major QTLs detected in the panel of 174 diverse oat accessions for GPC had two haplotypes: one favorable and another unfavorable for GPC. Haplotype G for QTL16 (QTL16-HAP-G) was present in 64 accessions with an average GPC ranging from 13.69% to 16.30% in different environments (Table 5), whereas haplotype A for QTL16 (QTL16-HAP-A) was presented in 77 accessions with a mean GPC of 10.72–13.04%. A significance test revealed that accessions with the haplotype G possessed a significantly higher GPC than accessions with the haplotype A (*p* < 0.001) in all the test environments (Table 5, Figure 5a). Hence, QTL16-HAP-G was considered as the favorable haplotype and QTL16-HAP-A as the unfavorable haplotype for oat GPC. Haplotype CAA in QTL11 (QTL11-HAP-CAA) was observed in 32 accessions that showed an average GPC of 13.51–16.51%, while haplotype TGC (QTL11-HAP-TGC) existed in 108 accessions showing a significantly lower average GPC of 11.33–13.47% (Table 5, Figure 5b). Therefore, QTL11-HAP-CAA was defined as the favorable haplotype and QTL11-HAP-TGC as the unfavorable haplotype. The frequency of the favorable haplotypes for both QTLs was found to be higher in hulless oat compared with hulled oats (Table 5, Figure 5c,d). Of the hulless oats, 86.67% harbored the QTL16-HAP-G favorable haplotype, while only 10.53% of the hulled oats carried it (Figure 5c). Similarly, more than half (51.67%) of the hulless accessions possessed the favorable haplotype for QTL11 (Figure 5d). In contrast, only one hulled oat (0.89%) was found to carry this allele. Interestingly, when analyzing the combined effect of the QTL11 and QTL16, the combination of the two favorable haplotypes showed a significantly higher GPC that the combination of the two unfavorable haplotypes. All these results suggest that favorable haplotypes for GPC are more common in hulless oats, and new varieties with high GPC could be developed by pyramiding favorable alleles of these loci.

### 2.5. Putative Candidate Genes and Annotations

The significant SNPs associated with GPC were used to pinpoint potential candidate genes using the recently annotated “Sanfensan” reference genome, as shown in Table 4. A total of 36 genes across different chromosomal regions were identified to be linked with significant QTLs. Of these genes, 22 had functional annotations, including *cytochrome P450-like* gene, MYB domain-containing proteins, and disease-resistance genes. The SNP, *S6C_285259972* on 6C which was found to be significantly associated with GPC in all tested environments and was located near the gene *A.satnudSFS6C01G004265*, while the three SNPs on 4C that were significantly associated with GPC in WJ20 and in the multi-environment model were situated close to gene *A.satnudSFS4D01G000779*. However, the functions of both genes have not yet been annotated.

## 3. Discussion

Groat protein content (GPC) is a key characteristic in oats and thoroughly investigating the phenotypic variation present in the existing oat germplasms is crucial for improving this trait. In the current study, the GPC of 174 diverse oat accessions from 42 countries or regions was evaluated through three field trials. These accessions were selected from a larger pool of 659 diverse oats (Diverse Oat Panel) that had been genotyped by GBS technology [28]. The selection of 174 oats from the Diverse Oat Panel was based on both the genetic diversity and origin (Figure S1), suggesting a diverse range of alleles among these accessions. The results show a significant level of phenotypic variation in GPC among the 174 oat accessions, with values ranging from 6.97% to 22.24% across the field trials. This range is wider than that of previous studies (10.9–20.8%) [7,15], indicating the existence of ample genetic variability in this collection.

Previous studies have shown that hulless oats have higher GPC than hulled oats [29,30]. However, these studies measured the GPC of hulled oats without the removal of the hulls, which can comprise over 30% of the total weight of the grain, and consist primarily of cellulose and hemicellulose [31]. In this study, the GPC of the hulled oats was assessed without the hulls, allowing for a direct comparison of GPC between the hulled and hulless oats. The result showed that, compared to the 114 hulled oats, the 60 hulless oats possessed a significantly higher mean GPC in all tested environments (Figure 2b, Table S2), thereby reinforcing the high GPC characteristic of hulless oats. Interestingly, no significant differences in GPC were observed between the landraces and cultivars in both hulled and hulless oats, suggesting that GPC has not been a primary focus in breeding programs for both types of oats.

Many studies have reported that GPC is influenced by the environment to a fairly high level [16,32,33]. This was confirmed by the contrasting results between Wenjiang in different years, as well as differences observed between Wenjiang and Chongzhou in 2020 in this study (Figure 1). Despite these findings, 11 lines were identified that consistently displayed high GPC in at least two field trials (Table 3). All these lines are hulless oats that originated from different provinces of China, represent diverse donors for high GPC, and thus could be used in breeding for GPC cultivars.

This study conducted a GWAS for oat GPC using phenotypic data in individual environments and BLUP values. A total of 27 non-redundant QTLs were identified (Table 4, Figure 4). Most of these QTLs had a relatively limited effect (R^2^ < 15%, Table S3). Correspondingly, the relative proportion of the environmental influence on these QTLs was high. This study identified only two QTLs that were stably detected in multiple environments, which is in line with a previous mapping study that found 15 QTLs associated with GPC but only three that were identified across all three environments [16]. Therefore, further studies conducted over a wider range of environments are necessary to validate the identified QTLs for oat GPC.

In this study, most of the identified QTLs were found on the C and D chromosomes, while only one QTL was located on chromosome 4A. This indicates that the C and D subgenomes possess a greater number of loci controlling the accumulation of groat content compared to the A-subgenome. This outcome is in accordance with previous studies that have shown that the C genome diploids and the CD genome tetraploid species tend to have higher levels of GPC than the A genome diploids and the AB genome tetraploids [34,35].

The availability of the high-quality oat reference genomes [2,28] enabled a comparison between known QTLs and those identified in this study. By aligning the DNA sequences of markers flanking the reported QTLs to the “Sanfensan” reference genome, the chromosomal locations of 13 known QTLs were determined; these were distributed across 10 chromosomes (Figure 4). However, there was limited overlap between the known and newly identified QTLs. This discrepancy may be due to the influence of the environment and the unique properties of the mapping populations used [22]. Despite this, the two QTLs on 4D (QTL11) and 6C (QTL16), which were consistently detected in various environments, have potential for future breeding efforts aimed at increasing GPC in oats.

Candidate gene predictions revealed potential associations between GPC and several important genes (Table 4). For example, MYB-related proteins were found to be associated with QTL4 on chromosome 2D, QTL19 and QTL21 on chromosome 6C. The MYB superfamily plays a variety of roles in almost all plant aspects [36]. Additionally, some QTLs were found to be associated with *cytochrome P450* and disease-resistance genes, which have also been recognized as potential candidate genes for GPC in wheat [37]. However, it is important to approach the proposed candidate genes with caution, as many genes could be associated with GPC due to the complex nature of the trait. The slow rate of linkage disequilibrium decay in oat chromosomes [28] also limited the mapping resolution to some extent.

This study found that the combination of the two superior haplotypes of the two major QTLs had a significantly higher GPC compared to the combination of the two inferior haplotypes. These findings suggest new varieties with high GPC can be developed by pyramiding these superior alleles. While previous studies have suggested a negative correlation between grain yield and GPC in cereal crops [33,38], some QTLs for GPC have been found to have no negative effect on grain yield [37,39,40], indicating the potential for improving GPC without compromising yield by incorporating these QTLs into new varieties. However, as the grain yield of the 174 oat accessions in this study was not investigated, the effects of the identified GPC QTLs on grain yield remain unknown. Further research is needed to estimate these effects, particularly for the two major QTLs for GPC, to provide valuable information on how to effectively utilize these genetic loci.

## 4. Materials and Methods

### 4.1. Plant Materials

In a previous study, a diverse panel of 659 oat accessions was collected and sequenced using the GBS strategy. This oat panel, referred to as the Diverse Oat Panel, contains 128 hulless oats from China, 145 oat lines nominated by breeders from North America and Europe, and 371 diverse accessions collected from 52 countries or regions [28]. Principal component analysis (PCA) revealed a weak population existed in this panel. Based on the PCA analysis, a subset of 174 oat lines was selected from the Diverse Oat Panel for the assessment of the GPC in multiple environments and GWAS analyses. This subset represented most of the genetic diversity and geographical distribution of the Diverse Oat Panel (Table S1, Figure S1) and was composed of 118 landraces, 51 modern cultivars, and five accessions of unknown improvement status from Shanxi province, China. Of the 174 oat accessions, 60 are hulless oats and 114 are hulled oats (Table S1). All the genotypes in the subset can survive to maturity in the Chengdu plain (103~105° E, 30~31° N), China.

### 4.2. Field Trials and Measurement of GPC

Oat accessions were planted at the Weijiang experimental farm of Sichuan Agricultural University in Wenjiang, Chengdu (103°51′ E, 30°43′ N) over two years (2019 and 2020), and at the Chongzhou Experimental Station of Sichuan Agricultural University in Chongzhou (103°38′ E, 30°32′ N) in 2020, referred to as WJ2019, WJ2020, and CZ2020, respectively. The experiments followed a randomized complete block design without replication at each location. Each plot comprised two 1.5 m rows with 30 cm inter-row spacing and 10 cm inter-plant spacing. Nitrogen and superphosphate fertilizers were applied one week prior to sowing at a ratio of 80 kg/ha. Field management, disease, pest, and weed control were carried out as needed. After maturity, the seeds from each oat accession were harvested separately by rows and dried in a forced-air oven at 50 °C to constant weight. Equal amounts of seed samples from each row were bulked to represent a single balanced sampling per line. Subsequently, seeds with similar sizes were selected for the measurement of crude protein content. Crude GPC was determined using a Kjeldahl 8400 nitrogen analyzer (FOSS, Hillerod, Denmark) and expressed as a percentage of dry weight. To make a comparison of the GPC between hulled and hulless oats, the hulls of the hulled oats were removed manually before analyses. Each sample from the three environments was analyzed three times to generate technical replicates, and the mean values among the technical replicates were used for further analysis.

### 4.3. Statistical Analysis

A frequency map was generated to display the distribution of GPC among the oat accessions in each environment. Descriptive statistics, including range, mean, standard deviation, and coefficient of variation, were summarized in R (v4.05) [41]. The significance of the differences in GPC between landraces and modern cultivars, as well as between hulless and hulled oats, was estimated using a two-tailed Student-t test. A general linear model was used to estimate the variance of the GPC by fitting genotype and environment into the model using the SPSS v27.0 software (SPSS, Chicago, IL, USA) with both genotype and environment considered as random effects. Since there was no replication in this study, the interactions between genotype and environment could not be estimated. Pearson’s correlation coefficients (r) of pairwise environments were calculated using the R package “corrplot v0.93” (https://github.com/taiyun/corrplot, accessed on 12 January 2023) to determine the consistency of GPC in different environments.

### 4.4. Genomic Data Analysis

In a prior study, 49,702 high-quality SNPs were identified after aligning the GBS reads from the 659 oat accessions in the Diverse Oat Panel to the “Sanfensan” reference genome [28]. The 49,702 sites of the 174 oat accessions used in this study were first filtered to (i) keep only SNPs with a minor allele frequency greater than 5%, (ii) exclude SNPs with more than 10% heterozygotes, and (iii) exclude SNPs with missing data (N) more than 20%. After the filtering steps, there were 38,313 SNPs kept in the genotypic matrix. Missing data were subsequently imputed using the Linkage Disequilibrium K-Number Neighbor Imputation (LDKNNi) method implemented in the TASSEL 5.0 software [42]. The resulting genotypic matrix was used in the following analyses.

### 4.5. Marker-Trait Association and Gene Annotations

Marker-trait associations were carried out for each trial separately, and also considering a multi-environment model. For multi-environment model analysis, best linear unbiased predictions (BLUPs) were estimated by the lme4 R package for use as the phenotypic input for the subsequent association analysis. The mixed linear model (MLM) method implemented in TASSEL 5.0 software was used to detect the association between markers and GPC, and the population structure and kinship information (K matrix) were taken care of to minimize false-positive associations. The population structure was represented by the first three principal components, which explained 34.77, 7.64, and 3.80% of the total variance, respectively, while the K matrix was estimated by using the centered identity-by-state method. Quantile-Quantile (Q-Q) plots and Manhattan plots were drawn using the R package “CMplot” [43]. Given the exploratory nature of this study, a relatively less-stringent *p*-value threshold of 0.001 (−log_10_ *p* = 3.0) was used to avoid removing true positive associations. Annotations for the associated markers for GPC were assigned based on the closest annotated gene identified near the SNP physical location using the “Sanfensan” reference genome, and protein functions were further explored through literature mining of annotated information.

### 4.6. Comparative Mapping

Several previous studies [16,17,18,19,20] have been conducted to identify QTLs for GPC in oat by using different RIL mapping populations. To compare the results of these studies with those of the present study, the chromosomal locations of these known QTLs were determined by aligning the available flanking markers to the reference genome using a blastn analysis. The best hits with a query cover > 90% and *e*-value < 10^−20^ were considered as their chromosomal locations of the flanking marker on the reference genome.

### 4.7. Estimate of Haplotye Effects

Haplotype analysis was carried out for the major QTLs detected in at least two environments using the LDBlockShow program [44]. The “favorable” haplotypes were defined to these have positive effects leading to higher GPC. Conversely, the alternative haplotypes with negative effects leading to lower GPC were considered as “inferior haplotypes”. The significance of the difference in GPC between accessions with superior alleles and inferior alleles was estimated by using a two-tailed Student’s *t*-test.

## 5. Conclusions

This study examined the GPC of 174 diverse oat accessions and revealed a wide range of variability in GPC. Hulless oats had a higher mean GPC compared to hulled oats in all field trials. A GWAS study identified 27 unique QTLs on 12 chromosomes that were associated with oat GPC. Two of them were stably detected in multiple environments. Genotypes with favorable alleles at these two major loci exhibited significantly higher GPC. In summary, this study highlights the genetic diversity and potential targets for enhancing the GPC in oats.

## Figures and Tables

**Table 1:** Summary of phenotypic analyses of groat protein content in oat accessions.

Environment *	Range (%)	Mean ± SD (%)	Coefficient of Variation (%)
WJ19	6.97–22.24	14.25 ± 3.06	21.48
WJ20	7.03–18.62	11.90 ± 2.44	20.51
CZ20	8.29–18.72	13.29 ± 2.55	19.16
BLUP	10.24–16.54	13.15 ± 1.43	10.86

* BLUP represents the best linear unbiased prediction.

**Table 2:** Analysis of variance of groat protein content.

Source	DF	SS	MS	*F*-Value	*p*-Value
Accessions	173	2298.10	13.28	3.11	<0.001
Environment	2	486.33	243.16	56.99	<0.001
Error	346	1476.23	4.28		

**Table 3:** List of oat accessions showing a consistent low or high protein content in field trials.

Accession ^a^	Origin	Improvement	Grain Type	Group ^b^	Protein Content (%) ^c^
Dominik	Germany	Cultivar	Hulled	Low	10.71 ± 2.40
Bullion	United Kingdom	Cultivar	Hulless	Low	8.88 ± 0.31
CN 21952	Ethiopia	Landrace	Hulled	Low	9.65 ± 0.81
CN 22244	Ethiopia	Landrace	Hulled	Low	9.33 ± 1.61
CN 53617	Tibet, China	Landrace	Hulless	Low	9.48 ± 1.26
CN 55126	Bulgaria	Landrace	Hulled	Low	10.25 ± 3.45
CN 69522	Ethiopia	Landrace	Hulled	Low	8.93 ± 0.82
CN 2841	Czech Republic	Landrace	Hulled	Low	9.77 ± 0.82
ZY001500	Hebei, China	Cultivar	Hulless	High	17.09 ± 4.52
ZY001746	Inner Mongolia, China	Cultivar	Hulless	High	17.22 ± 0.83
ZY000054	Hebei, China	Landrace	Hulless	High	17.18 ± 2.32
ZY001821	Shanxi, China	Cultivar	Hulless	High	17.44 ± 4.30
ZY001960	Shanxi, China	Landrace	Hulless	High	16.25 ± 3.42
ZY002019	Shanxi, China	Landrace	Hulless	High	18.13 ± 1.73
ZY000100	Inner Mongolia, China	Landrace	Hulless	High	16.52 ± 3.42
20130090	Shanxi, China	Unknown	Hulless	High	15.90 ± 1.21
ZY000264	Inner Mongolia, China	Cultivar	Hulless	High	15.66 ± 3.32
ZY000517	Shanxi, China	Landrace	Hulless	High	15.95 ± 2.91
ZY000584	Gansu, China	Landrace	Hulless	High	16.34 ± 1.83

^a^ Seeds with accession number starting with characters “CN” were provided by the Plant Gene Resources of Canada (PGRC). The remaining accessions were provided by Xichang Academy of Agricultural Sciences, Chinese Crop Germplasm Resources Information System (CGRIS) or Shanxi Academy of Agricultural Sciences. ^b^ Accessions that showed a consistent low (bottom 20) or high (top 20) protein content in at least two environments were grouped as low or high genotypes, respectively. ^c^ Protein content was averaged across three environments and displayed as mean±standard deviation value.

**Table 4:** Significant loci and their chromosomal locations, corresponding proteins and possible function elucidated based on the gene annotation using oat reference annotation database.

QTL	Chromosome	SNP	Gene ID	Dist. From SNP (in Bp)	Annotation
QTL1	1D	S1D_250255160	*A.satnudSFS1D01G005211*	+42,460	-
QTL2	1D	S1D_442130558	*A.satnudSFS1D01G001001*	+4643	Omega-amidase, chloroplastic
QTL3	2C	S2C_532984931	*A.satnudSFS2C01G004386*	+11,305	BTB/POZ and MATH domain-containing protein 2-like
QTL4	2D	S2D_170852683	*A.satnudSFS2D01G003434*	+33,688	Transcription factor MYB39-like
QTL5	2D	S2D_475816930	*A.satnudSFS2D01G006139*	+265,929	-
		S2D_476389956	*A.satnudSFS2D01G006142*	−112,580	Dirigent protein 7-like
		S2D_479293592	*A.satnudSFS2D01G006174*	+2123	Gibberellin 2-beta-dioxygenase 8-like
		S2D_481506595	*A.satnudSFS2D01G006200*	−78,257	-
		S2D_481506711	*A.satnudSFS2D01G006200*	−78,141	-
		S2D_483972532	*A.satnudSFS2D01G006225*	−272,059	Flowering-promoting factor 1-like protein 4
		S2D_484596713	*A.satnudSFS2D01G006226*	−168,983	Fasciclin-like arabinogalactan protein 14
		S2D_485481234	*A.satnudSFS2D01G006232*	−194,688	Transcription factor 3C polypeptide 5-like
QTL6	3C	S3C_539312598	*A.satnudSFS3C01G003718*	+6886	-
QTL7	3D	S3D_93982802	*A.satnudSFS3D01G001144*	+50,123	-
QTL8	3D	S3D_323864116	*A.satnudSFS3D01G002332*	+23,877	Zinc finger protein BRUTUS-like
		S3D_329141631	*A.satnudSFS3D01G002420*	+22,075	Ribosomal protein L36a
QTL9	3D	S3D_404716753	*A.satnudSFS3D01G003689*	−2211	Ribosomal protein L18-2
QTL10	4A	S4A_435900993	*A.satnudSFS4A01G006735*	+7552	Axoneme-associated protein mst101(2)-like
QTL11	4D	S4D_422496061	*A.satnudSFS4D01G000876*	+41,624	Probable leucine-rich repeat receptor-like protein kinase
		S4D_425631870	*A.satnudSFS4D01G000779*	−3731	-
		S4D_425631876	*A.satnudSFS4D01G000779*	−3725	-
		S4D_425632011	*A.satnudSFS4D01G000779*	−3590	-
QTL12	5C	S5C_42034228	*A.satnudSFS5C01G000409*	+28,493	-
QTL13	6C	S6C_112862861	*A.satnudSFS6C01G002256*	+4234	Putative disease resistance RPP13-like protein 3
QTL14	6C	S6C_154091753	*A.satnudSFS6C01G003130*	−39,300	-
QTL15	6C	S6C_238593165	*A.satnudSFS6C01G003976*	−3784	-
QTL16	6C	S6C_285259972	*A.satnudSFS6C01G004265*	−9039	-
QTL17	6C	S6C_516086587	*A.satnudSFS6C01G005207*	−58,400	Polymerase 2-A
QTL18	6C	S6C_522114339	*A.satnudSFS6C01G005237*	+64,919	Pop guanine nucleotide exchange factor 14
QTL19	6C	S6C_528407767	*A.satnudSFS6C01G005283*	+68,520	-
		S6C_531364922	*A.satnudSFS6C01G005292*	−405,403	Myb-related protein Zm1-like
QTL20	6C	S6C_540012059	*A.satnudSFS6C01G005352*	+34,825	Cytosolic sulfotransferase 5
QTL21	6C	S6C_583370024	*A.satnudSFS6C01G005704*	+94,938	R2R3-MYB protein
QTL22	6D	S6D_26224217	*A.satnudSFS6D01G000303*	−2965	-
		S6D_26224354	*A.satnudSFS6D01G000303*	−2828	-
QTL23	6D	S6D_196278046	*A.satnudSFS6D01G001314*	+64,283	-
QTL24	7C	S7C_176236166	*A.satnudSFS7C01G002963*	+13,241	Cytochrome P450 94B3-like
		S7C_176236263	*A.satnudSFS7C01G002963*	+13,338	Cytochrome P450 94B3-like
QTL25	7C	S7C_191492694	*A.satnudSFS7C01G003117*	+19,910	-
QTL26	7C	S7C_413114334	*A.satnudSFS7C01G003963*	−636,910	-
QTL27	7D	S7D_455264275	*A.satnudSFS7D01G004588*	+3682	Hydrophobic protein RCI2A-like

**Table 5:** Average protein contents of accessions harboring favorable and unfavorable haplotypes in different environments, and the frequency of the favorable and unfavorable haplotypes in hulless and hulled oats.

QTL	Haplotype	Protein Content (%)				Hap Frequency (%) ^#^	
		WJ19	WJ20	CZ20	BLUP	Hulless	Hulled
QTL16	HAP-G	16.30	13.67	15.09	14.43	86.67	10.53
	HAP-A	13.04	10.72	12.11	12.34	10.00	62.28
QTL11	HAP-CAA	16.52	13.51	15.62	14.56	51.67	0.88
	HAP-TGC	13.48	11.33	12.77	12.73	33.33	77.19
QTL16 + QTL11	HAP-CAA + HAP-G	16.81	13.85	15.84	14.75	43.33	0
	HAP-CAA + HAP-A	15.73	11.41	15.08	13.78	5.00	0.89
	HAP-TGC + HAP-G	15.83	13.13	14.27	14.03	28.33	8.77
	HAP-TGC + HAP-A	12.52	10.57	12.16	12.20	5.00	46.49

^#^ The frequency of each haplotype or haplotype combination in hulless and hulled oats was determined as the proportion of accessions that carried the haplotype or haplotype combination.

## Data Availability

The data presented in this study are included in the article/Supplementary Materials, further inquiries can be directed to the corresponding authors.

## Supplementary Materials

The following supporting information can be downloaded at: https://www.mdpi.com/article/10.3390/ijms24065581/s1.
