# Supplementary figures and images for: Genome-Wide Association Mapping of QTL Underlying Groat Protein Content of a Diverse Panel of Oat Accessions
Source: Int J Mol Sci. 2023 Mar 15;24(6):5581. doi: 10.3390/ijms24065581 (PMC10053717; doi:10.3390/ijms24065581)

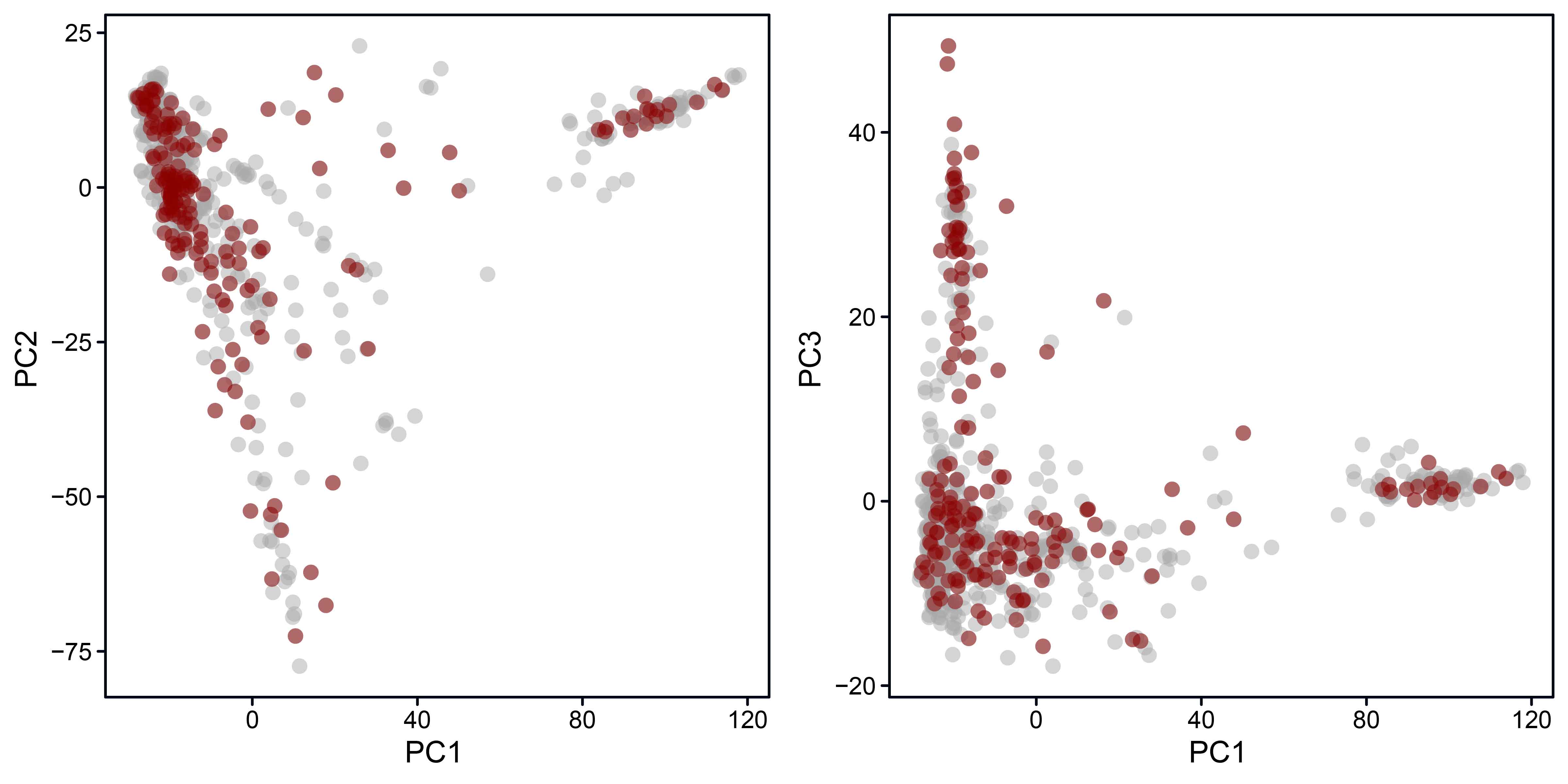

Supplement: Supplementary file 1 [file ijms-24-05581-s001.zip › Figure S1.jpg]
